# The application of extended reality in cardiac surgery: potential implications for low- and middle-income countries

**DOI:** 10.1097/JS9.0000000000000082

**Published:** 2023-01-12

**Authors:** Yumna Khabir, Mushkbar Khan, Urooj Iqbal

**Affiliations:** Department of Medicine, Dow University of Health Sciences, Karachi, Sindh Province, Pakistan

*Dear Editor,*

Cardiovascular diseases (CVDs) are the leading cause of death worldwide and impose a significant burden. Based on the 2022 Global Burden of Disease Statistics by the American Heart Association, in 2020, ∼19.1 million deaths were attributed to CVD globally^1^. An estimated 17.9 million people died from CVDs in 2019, representing 32% of all global deaths, of which three-quarters of CVD deaths occurred in low- and middle-income countries (LMICs)^2^. As a result of cutting-edge technologies and expensive medications, the prevalence of CVDs is decreasing in high-income countries. However, it is still a considerable threat to LMICs. To combat CVDs and their prevalence in LMICs, it is advised that supportive strategies and better healthcare intervention systems, like cardiac surgery, be used.

Due to the reliance on multiple health services, cardiac surgery is an expensive and complex intervention. However, despite the high procedural costs, it is still cost-effective due to its significant impact on individuals and populations. Pediatric cardiac surgery in LMICs can be cost-effective at $171 per disability-adjusted life year, which is more favorable than many common global public health interventions, such as oral rehydration therapy for diarrhea and HIV/AIDS treatment^3^.

Recent years have seen computing devices emerge that can immerse users in a digital reality or overlay digital information onto physical reality. Many terms are used to describe and classify these devices, some with overlapping definitions or ambiguous interpretations. For this reason, the term “extended reality” (XR) has recently gained favor as an umbrella term that encompasses all of AR (augmented reality), VR (virtual reality), and MR (mixed reality)^4^.

While VR displays are suitable for educational or preprocedure applications, 3D AR displays are generally better suited during procedures as they do not obscure the physician’s vision. Recent studies have used this approach to create a 3D image of a myocardial scar using late gadolinium enhancement with the Microsoft HoloLens^4^. The mapping specialists and operators who used the visualization praised its usefulness during the intervention. A similar experiment has been conducted using the RealView Holographic Display system, which projects holographic images without requiring a headset^4^.

The cost of implementing XR in healthcare settings has been a significant barrier to its use in surgery since the technology’s conception. This is especially true for cardiac surgery, which demands high fidelity and accuracy from XR simulations regardless of their intended use^5^. Consequently, the technology is not cost-effective because the computer’s processing power alone is inadequate for these simulations. However, the cost barriers to using XR in a surgical setting have decreased over the past decade as less expensive technology with significantly stronger processing power has entered the commercial market, and the opportunities for XR to enhance patient safety, surgical training, and audit quality have become clear. In particular, costs associated with implementing XR technology in a surgical setting are decreasing thanks to widely applicable commercial hardware use and adaptation. An example is the recently developed VR headsets, which offer realistic hand interactions and high-quality visuals.

With the help of XR, experienced cardiac surgeons in one region of the world could instruct cardiac surgeons and residents to perform those intricate procedures in different parts of the world, saving money and time on travel. This would allow people to receive procedures in their country rather than traveling abroad and assist LMICs in learning from surgeons in nations with better surgical systems. This is a step toward achieving the primary goal of health policymakers and healthcare planners, which is to eliminate health disparities between individuals at opposite ends of socioeconomic gradients.

The XR hardware landscape is changing rapidly. Future hardware advances should improve visual realism and user comfort. Incorporating haptic feedback into XR systems may be an important breakthrough for interventional procedures. This, along with technologies such as 5G connectivity going mainstream as well as artificial intelligence platforms in healthcare and quantum computing, holds promise for the future outlook of medicine in LMICs.

## Ethical approval

No ethical approval was required for this search.

## Sources of funding

None.

## Authors’ contribution

Y.K.: conceptualization, literature review, manuscript writing, and reviewing. M.K.: conceptualization, literature review, and manuscript writing. U.I.: literature review and manuscript writing.

## Conflicts of interest disclosure

The authors declare that they have no financial conflict of interest with regard to the content of this report.

## Research registration unique identifying number (UIN)

Not applicable.

## Guarantor

Yumna Khabir, Mushkbar Khan, Urooj Iqbal.
